# Lipogenic Gene Single Nucleotide Polymorphic DNA Markers Associated with Intramuscular Fat, Fat Melting Point, and Health-Beneficial Omega-3 Long-Chain Polyunsaturated Fatty Acids in Australian Pasture-Based Bowen Genetics Forest Pastoral Angus, Hereford, and Wagyu Beef Cattle

**DOI:** 10.3390/genes13081411

**Published:** 2022-08-08

**Authors:** John R. Otto, Felista W. Mwangi, Shedrach B. Pewan, Oyelola A. Adegboye, Aduli E. O. Malau-Aduli

**Affiliations:** 1 Animal Genetics and Nutrition, Veterinary Sciences Discipline, College of Public Health, Medical and Veterinary Sciences, Division of Tropical Health and Medicine, James Cook University, Townsville, QLD 4811, Australia; 2 National Veterinary Research Institute, PMB 01, Vom 930001, Plateau State, Nigeria; 3 Public Health and Tropical Medicine Discipline, College of Public Health, Medical and Veterinary Sciences, Division of Tropical Health and Medicine, James Cook University, Townsville, QLD 4811, Australia

**Keywords:** SNP, health-beneficial omega-3 fatty acids, lipogenic genes, intramuscular fat, fat-melting point, NGS

## Abstract

This study used targeted sequencing aimed at identifying single nucleotide polymorphisms (SNP) in lipogenic genes and their associations with health-beneficial omega-3 long-chain polyunsaturated fatty acids (n-3 LC-PUFA), intramuscular fat (IMF), and fat melting point (FMP) of the *M. longissimus dorsi* muscle in Australian pasture-based Bowen Genetics Forest Pastoral Angus, Hereford, and Wagyu cattle. It was hypothesized that SNP encoding for the fatty acid-binding protein 4 (FABP4), stearoyl-CoA desaturase (SCD), and fatty acid synthase (FASN) genes will be significantly associated with health-beneficial n-3 LC-PUFA and the meat eating quality traits of IMF and FMP in an Australian pasture-based beef production system. Two SNP mutations, g.21267406 T>C and g.21271264 C>A, in the SCD gene were significantly (*p* < 0.05) associated with IMF, FMP, oleic acid (18:1n-9), linoleic acid (LA) 18:2n-6, alpha-linolenic acid (ALA) 18:3n-3, eicosapentaenoic acid (EPA) 20:5n-3, docosahexaenoic acid (DHA) 22:6-n-3, and docosapentaenoic acid (DPA) 22:5n-3. Significant positive correlations (*p* < 0.05) between FASN SNP g. 50787138 A>G and FMP, 18:1n-9, ALA, EPA, DHA, DPA, and total n-3 LC-PUFA were also detected. An SNP (g.44678794 G>A) in the FABP4 gene was associated with FMP. These results provide significant insights into the contributions of lipogenic genes to intramuscular fat deposition and the biosynthesis of health-beneficial n-3 LC-PUFA. The findings also unravel the potential use of lipogenic gene polymorphisms in marker-assisted selection to improve the content of health-promoting n-3 LC-PUFA and meat eating quality traits in Australian pasture-based Bowen Genetics Forest Pastoral Angus, Hereford, and Wagyu beef cattle.

## 1. Introduction

Consumers demand healthier, safer, and more consistent beef of high eating quality [1,2]. Currently, the Australian and global beef industry, supported by research bodies, has shifted its attention to finding ways of producing meat of consistent eating quality and health-promoting ≥C20 omega-3 long-chain polyunsaturated fatty acids (n-3 LC-PUFA) that play essential roles in human disease prevention [3,4]. Intramuscular fat content (IMF), fat melting point (FMP), and ≥C20 n-3 LC-PUFA composition are associated with consumer satisfaction and key eating quality attributes in the Australian, American, and Japanese beef industries because of their associations with taste, smell, texture, tenderness, flavor, and juiciness [1,5,6,7]. The essential omega-3 fatty acids of interest include alpha-linolenic acid (ALA), eicosapentaenoic acid (EPA), docosahexaenoic acid (DHA), and docosapentaenoic acid (DPA) [8,9]. However, meat also contains saturated fatty acids (SFA) such as palmitic, myristic, and lauric acids, which are the main contributors to blood plasma cholesterol, and low-density lipoproteins (LDL), two key concerns for beef consumers [10].

The composition of n-3 LC-PUFA in the muscle tissue is influenced by animal diet, age, production system, and genetics [3,11]. Several studies utilized dietary means to manipulate fat deposition and fatty acid composition [12,13,14,15,16]. However, the results obtained were variable and contentious due to differences in breed, diet, dosage, production system, and rumen biohydrogenation [17,18]. Since lipogenic genes are involved in fatty acid metabolism within the muscle tissue [19,20], genetic selection and breeding present an excellent opportunity for a long-term, permanent, and cumulative approach to improving beef n-3 LC-PUFA content and eating quality [21] because of additive genetic variation for fatty acids in most beef cattle breeds [22]. However, measuring these meat quality traits is expensive as data are acquired after the animal is dead. Furthermore, current technologies developed to measure meat eating quality characteristics have low precision and high inconsistency, characterized by a wide divergence between calibration and validation data [23]. Such inaccuracies and low precision in the measurement of meat quality attributes cost the Australian beef industry AUD 130 million per year [23]. Other previously proposed methods for predicting IMF and fatty acid composition in beef include the Bayesian-based Single Nucleotide Polymorphisms (SNP) Best Linear Unbiased Predictor, Least Absolute Shrinkage and Selection Operator, and X-ray absorptiometry scanner methods [24,25,26,27,28]. Most of these predictive models are still under study [22] and can potentially increase genetic gains for fat deposition and fatty acid composition. However, they differ widely in their assumption of the genetic model associated with quantitative traits and present precision problems due to low accuracy [29].

Recent technological advancements in genotyping and next-generation sequencing (NGS) have made it possible and affordable to accurately generate large-scale functional genomic data from livestock [30]. Thus, NGS has enabled a novel, efficient and cost-effective means of sequencing candidate genes, allowing for an in-depth interrogation of their organization, structure, function, and complexities to better understand their role in fat deposition and fatty acid composition in animal tissues [31]. NGS genotyping has been used in animal and human studies to detect and diagnose diseases [32,33] and in targeted sequencing of meat quality traits in sheep [9] and tropical beef cattle [34].

SCD, FABP4, and FASN are lipogenic genes known to influence fat deposition and fatty acid metabolism in meat and adipose tissues [6]. SNP identification may further enhance our current understanding of the contribution of lipogenic genes to intramuscular fat deposition and fatty acid composition in the muscle tissue of diverse beef cattle breeds [35,36,37,38,39]. The identified SNP can be used effectively for marker-assisted selection to improve health-beneficial n-3 LC-PUFA and meat eating quality traits.

Identified SNP in FASN and FABP4 genes have been associated with marbling score and meat quality grade in Angus, Hereford, Holstein, Hanwoo, Yanbian Yellow, Qinchuan, and Brangus beef cattle [11,35,40,41,42,43]. Likewise, SCD SNP were reported to be associated with fat deposition and fatty acid composition in beef cattle breeds [44,45,46,47]. This study aims to investigate the associations between identified SNP in SCD, FASN, and FABP4 lipogenic genes with health-beneficial n-3 LC-PUFA, IMF, and FMP in the *M. longissimus dorsi* muscles of Australian pasture-based Bowen Genetics Forest Pastoral Angus, Hereford, and Wagyu beef cattle. We hypothesized that significant associations exist between SNP in *SCD* (stearoyl-CoA desaturase), *FASN* (fatty acid synthase), and *FABP4* (fatty acid binding protein 4) genes with *M. longissimus dorsi* muscle n-3 LC-PUFA and meat eating quality traits under an Australian pasture-based beef production system.

## 2. Materials and Methods

### 2.1. Animal Ethics

This project was approved by the James Cook University Animal Ethics Committee (Approval Number A2724), under the Animal Care and Protection Act for the Australian Code for the Care and Use of Animals for Scientific Purposes.

### 2.2. Animals and Management

The animals used in this study were Angus, Hereford, and Wagyu raised and maintained under the same on-farm management at the Bowen Genetics Forest Pastoral stud, Barraba, New South Wales, Australia. A total of 127 yearling Angus, Hereford and Wagyu cattle were utilized for this study. The animals were fed on ryegrass pasture, kept in the same herd, and maintained under the same management routine. This cohort of beef cattle was of similar average age (10–11 months), body condition score (3.0–3.5), and liveweight (450–455 kg) to minimize any phenotypic variation and potential confounding factors.

### 2.3. Muscle Biopsy Sampling Procedure

Based on the procedure first described by Malau-Aduli et al. [48], the animals were gently restrained in a crush, and the hair around the 12th and 13th ribs was shaved with clippers. An alcohol/chlorhexidine disinfectant solution was used to clean the shaved surgical site. Approximately 20 mL of a local anesthetic, lignocaine, was injected at the surgical site before 2 g of muscle biopsy was sampled. The skin was closed with non-absorbable braided suture material to close the incision firmly, and antibiotics were administered to prevent secondary infection. The muscle biopsy samples were immediately placed in a plastic bag on dry ice, flushed with nitrogen gas, transferred into a mobile refrigerator, and stored at −20 °C awaiting further analysis in the laboratory. Each animal was checked daily to ensure that there were no complications. After ten days, the sutures were removed, and the animals were placed in larger yards for further observation for 2–3 days before returning to the herd. The muscle biopsies were analyzed for IMF, FMP, and fatty acid composition.

### 2.4. Determination of Fatty Acid Profile, Intramuscular Fat, and Fat Melting Point

Procedures for the determination of fatty acid profile, IMF, and FMP had previously been described extensively [9,21,49], but for the purposes of clarity, they are repeated herein.

#### 2.4.1. Determination of Intramuscular Fat

Briefly, the muscle sample was homogenized, and 1g transferred to a labelled 50 mL plastic tube containing 20 mL of chloroform: methanol (2:1) solvent and shaken vigorously for 5 min. A filter paper was used to collect the filtrate in another labelled 50 mL tube. Approximately 5 mL of 10% KCl was added to the filtrate to precipitate and separate the inorganic and lipid fractions into two distinct layers. The upper inorganic layer was removed and discarded, while the lower lipid layer was transferred into a clean, dry, pre-weighed and labelled ceramic crucible and evaporated in a laminar fume hood over a heating block. The crucible was cooled and further dried in a desiccator for 10–20 min before it was re-weighed. Samples were analyzed in duplicate to allow for replication and reproducibility. Intramuscular fat percentage was calculated as: [(Final crucible weight) − (Initial crucible weight)/(Initial sample weight)] × 100.

#### 2.4.2. Determination of Fat Melting Point

The crucible containing the extracted IMF was placed in an oven at 100 °C for about 1–2 min to melt the fat. Using air suction, the melted fat was sucked into a thin capillary tube and placed in a refrigerator for about 10 min for the fat to solidify. The fat level in the capillary tube was marked with an indelible pen. The capillary tube was attached to a thermometer and vertically suspended in a beaker containing 80 mL of cold water, gradually heated over a heating block, and closely observed until the fat melted and “slipped” (rose above the mark) within the capillary tube. The temperature at which this slip occurred was recorded as the fat melting point. Samples were analyzed in duplicate to allow for replication and reproducibility.

#### 2.4.3. Determination of Fatty Acid Composition

Total lipids in 1 g of un-homogenized muscle tissue samples were extracted overnight. The original phase was a single-phase overnight extraction utilizing CHCl3:MeOH:H2O (1:2:0.8 *v*/*v*). The second segment involved phase separation with the addition of CHCl3:saline Milli-Q H2O (1:1 *v*/*v*) followed by rotary evaporation of the lower chloroform phase at 40 °C to acquire total lipids. The extracted cumulative lipids were separated into lipid classes by thin-layer chromatography (TLC) using 100 mL of the lipid extract reconstituted in hexane. The extract was marked onto silica gel G plates (200 × 200 × 0.25 mm^3^) using a micropipette. The TLC plate was developed in an acetone/petroleum ether (1:3.147 *v*/*v*) solvent system in a tank comprising a few crystals of butylated hydroxytoluene (BHT) to hinder oxidation. Triacylglycerols, cholesterol and free fatty acids migrated, while phospholipids remained at the origin of the plate. The phospholipids were scraped off the plate into clean screw-capped test tubes for transmethylation and eventual computation of the lipid conversion factor (LCF) of 0.912 based on g fatty acids/g total lipids (0.083 for phospholipids, 0.829 for triacylglycerols and 0% for cholesterol since cholesterol does not have any fatty acids). An aliquot from each total lipid extract was utilized for transmethylation with MeOH:CHCl3:HCl (10:1:1 *v*/*v*) for 2 h at 80 °C. Fatty acid methyl esters (FAME) were extracted thrice using hexane:CHCl3 (4:1 *v*/*v*). A known concentration of an internal standard (C19:0) was added in a 1500 μL vial encompassing the extracted FAME. The FAME was analyzed on a 7890B gas chromatograph (Agilent Technologies, Palo Alto, CA, USA) furnished with an EquityTM -1 fused 15 m silica capillary column with 0.1 mm internal diameter and 0.1 μm film thickness (Supelco, Bellefonte, PA, USA), a flame ionization sensor, a split/splitless injector and an Agilent Technologies 7683 B Series autosampler. The gas chromatograph settings were splitless mode injection; carrier gas He; original oven temperature 120 °C and then increased to 270 °C at flow rates of 10 °C/min and to 310 °C at 5 °C/min. The Agilent Technologies ChemStation software (Palo Alto, CA, USA) was used to measure fatty acid peaks. The fatty acid identities were established using a Finnigan Thermoquest GCQTM GC/MS fitted with an on-column injector and Thermoquest Xcalibur software (Austin, TX, USA). Fatty acid percentages were calculated as follows: FA % = [(individual fatty acid area) × (100)]/(sum total area of fatty acids). Fatty acid contents were calculated as follows: FA mg/100 g = (Total lipid) × (LCF [0.912]) × ([%FA]/100) × 1000, where 0.912 was the resultant lipid conversion factor.

### 2.5. Blood Collection and Genomic DNA Isolation

Ten mL of blood samples were collected and stored at −80 °C until further analysis. Genomic DNA was extracted from 2 mL of blood using NucleoSpin Blood Kit (Macherey-Nagel GmbH & Co., Duren, Germany) following the procedures prescribed by the manufacturer. The yield and purity of the extracted genomic DNA was quantified using QuantiFluor^®^ dsDNA System (Promega, Madison, WI, USA).

### 2.6. Primer Design, Long-Range Amplification of Target Genes, PCR Clean-Up Products, Library Preparation, Sequencing, and Bioinformatics Analysis

**Figure 1:**
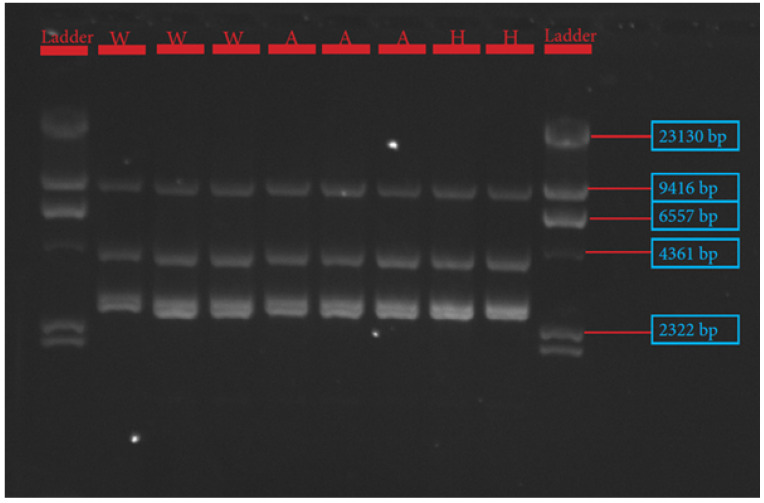
Gel image of stearoyl Co-A desaturase (SCD) gene fragments 1–4.

**Figure 2:**
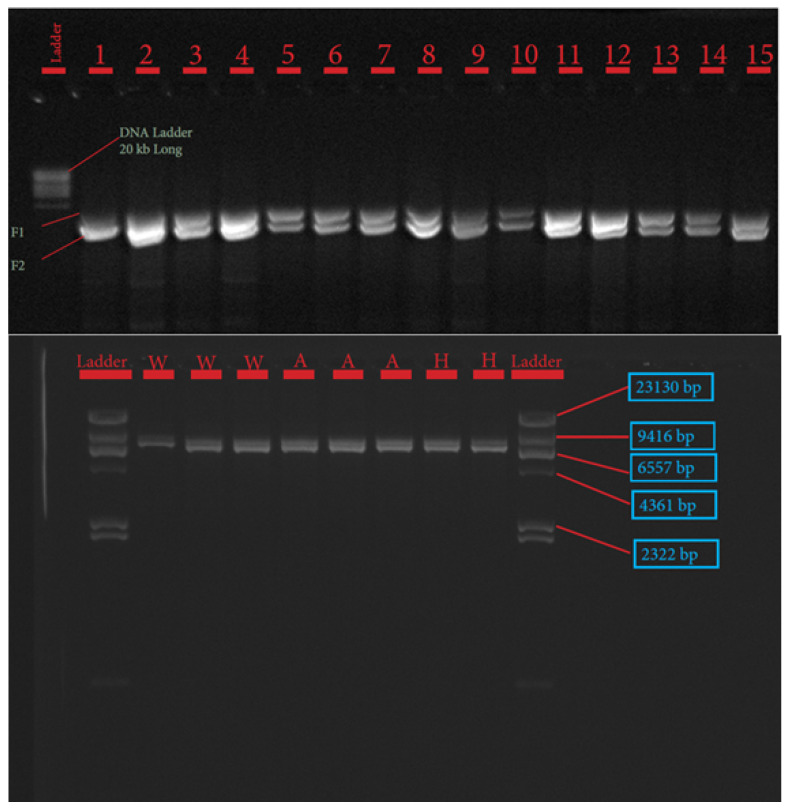
Gel image of fatty acid synthase (FASN) gene fragments 1–2.

**Figure 3:**
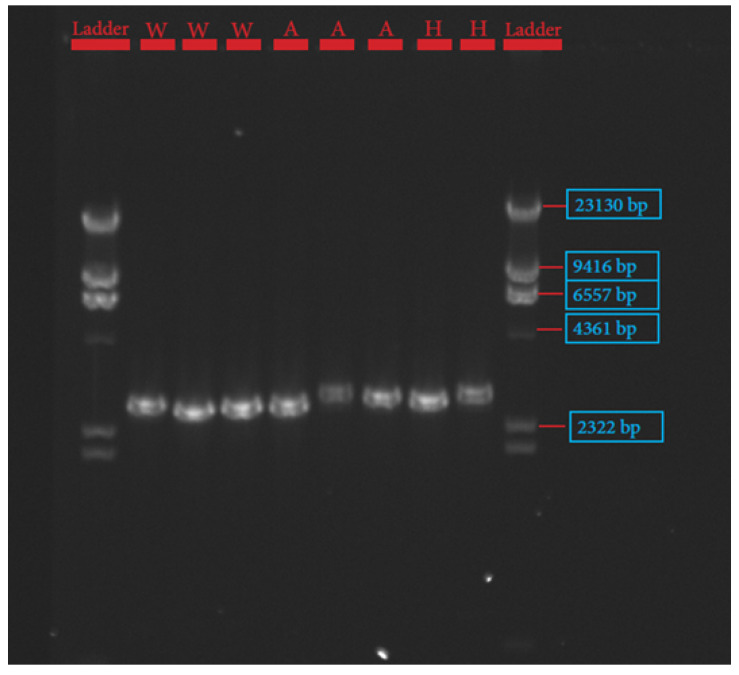
Gel image of fatty acid binding protein-4 (FABP4) gene.

The procedures for primer design, PCR amplification, library preparation, SNP sequencing, bioinformatics, and data analysis have been described extensively by Pewan et al. [50]. The single coding sequence of the Hereford breed deposited in the GenBank database for FASN, FABP4, and SCD genes with Accession Numbers NC_037346.1, NC_037353.1 and NC_037341.1, respectively, was utilized as a genotyping reference. All primer sequences used for amplifying target genes (Table 1) were designed using Geneious Prime software, synthesized at Integrated DNA Technologies Pte. Ltd., Singapore (itddna.com), and gel images of the resulting PCR products (Figure 1, Figure 2 and Figure 3) confirmed successful amplification.

#### 2.6.1. Long-Range PCR

Briefly, because of the different fragment lengths and DNA composition, it was necessary to use three different long-range PCR approaches to amplify the FASN, FABP4, and SCD genes. During optimization, all three approaches were tested for all three genes, but only the best performing combinations were utilized.

#### 2.6.2. FASN Gene

TakaRa PrimeSTAR GXL Master Mix (TaKaRa Bio Inc., Kusatsu, Shiga, Japan) was used to perform FASN PCR amplification assay. PCR reaction assay was set up in a total volume of 50 µL containing 10 µL of 5× TakaRa PrimeSTAR GXL Buffer, 200 µM of TaKaRa dNTP Mixture, 1.25 units of TaKaRa PrimeSTAR GXL DNA Polymerase, 0.2 µM of each primer and 100 ng of DNA template. PCR was performed in a SimpliAmp™ Thermal Cycler (Thermofisher Scientific, Melbourne, Australia), in a 2-step protocol using the following conditions: 98 °C initial denaturation for 1 min (1 cycle); 98 °C denaturation for 10 s; 68 °C annealing/extension for 10 min for 30 cycles. PCR success was checked in a 0.8% agarose gel electrophoresis image, as depicted in Figure 1.

#### 2.6.3. FABP4 and SCD Genes

Platinum™ SuperFi™ II PCR Master Mix (Thermofisher Scientific, Melbourne, Australia) and Hot Start II High-Fidelity PCR Master Mix (Thermofisher Scientific, Melbourne, Australia) were used to perform FABP4 and SCD PCR amplification assay under the same PCR conditions described below. The amplification reactions were performed in a total volume of 50 µL containing 25 µL of 2× Platinum™ SuperFi™ II PCR Master Mix or Phusion Hot Start II High-Fidelity PCR Master Mix (Thermofisher Scientific, Melbourne, Australia), 0.5 µM of each primer, and 100 ng of DNA template. PCR was performed in a SimpliAmp™ Thermal Cycler (Thermofisher Scientific, Melbourne, Australia), in a three-step protocol, using the following conditions: 98 °C initial denaturation in 1 min (1 cycle); 98 °C denaturation for 15 s; 60 °C (FABP4)/and 65 °C (SCD) annealing for 15 s; 72 °C extension for 9 min; 72 °C final extension for 9 min; 4 °C hold for 35 cycles. PCR success was checked in a 0.8% agarose gel electrophoresis image, as depicted in Figure 2 and Figure 3.

#### 2.6.4. PCR Clean-Up

PCR products were cleaned using Sera-Mag™ SpeedBeads (Merck KGaA, Darmstadt, Germany) in a Zephyr NGS Workstation (Caliper Lifesciences, Perkin-Elmer; Waltham, MA, USA) and quantified using a Promega dsDNA Quantifluor System Kit (Ref: E2670, 00002484139; Madison, WI, USA) on an Enspire Workstation (Perkin-Elmer; Waltham, MA, USA). The PCR products of all fragments were pooled and normalized to 0.2 ng/µL with 10 mM Tris-HCl (pH 8.0) for library preparation, and final accuracy checks of concentration were performed using the Illumina Nextera XT DNA Library Prep Kit (Illumina, San Diego, CA, USA).

#### 2.6.5. Library Preparation, Quantification, Normalization, and Sequencing

Libraries were prepared using Nextera XT DNA Library Prep kit (Illumina, San Diego, CA, USA) following the manufacturer’s protocols using the recommended input of 5 µL of 0.2 ng/µL gDNA per sample. This was followed by Sera-Mag™ SpeedBeads purification using 0.6× beads and two washes using 80% ethanol to select fragments >250 bp and remove unincorporated adapters. Each DNA library fragment size and concentration were determined using Agilent High Sensitivity D5000 reagents and ScreenTape on the Tape Station 4200 Instrument (Agilent Technologies, Santa Clara, CA, USA) according to the Agilent assay guide. Additionally, all individual libraries were quantified using QuantiFluor^®^ dsDNA System (Promega, Madison, WI, USA) to give an additional concentration estimate. The resultant size and concentration data from Tape Station and Quantifluor system were used to normalize each library to 4 nM by diluting with 10 mM Tris-HCl (pH 8.5) prior to pooling all samples. An equal volume of 5 µL was pooled and sequenced on an Illumina MiSeq benchtop sequencer (Illumina, Inc., San Diego, CA, USA), using a 500-cycle MiSeq Reagent Nano Kit v2 with a 10 pM input and 10% PhiX spike-in.

#### 2.6.6. Bioinformatics and Next Generation Sequencing Data Analysis

Genomic data analysis was performed using the commercial bioinformatics program Geneious Prime software program 2022 v.1.1 (http://www.geneious.com, accessed on 15 January 2022) to analyze the sequences. The following reference sequences deposited in the NCBI database were used for comparative analysis: NC_037346.1, NC_037353.1, and NC_037341.1 for FASN, FABP4, and SCD genes, respectively. NGS sequenced data were retrieved from Illumina Dashboard-BaseSpace Sequence Hub (https://basespace.illumina.com/dashboard (accessed on 15 January 2022)) as paired read data in two separate forward and reverse read lists in FASTQ format. The retrieved raw reads were subjected to quality control measures. Reads were trimmed and adapters removed using the BBDuk trimmer in Geneious Prime 2022 v.1.1 with the default setting for paired-end reads. The Quality (Q) value of Phred score was set at 20 to improve sequenced data and increase the likelihood of calling true SNPs to 99%. Short reads with a minimum length of 20 bp were discarded, resulting in clean reads. Low coverage regions were excluded when calling SNPs using the Annotate and Predict→Find Low/High Coverage. The reads were mapped to reference in Geneious. The reference sequences were retrieved from the NCBI database (Genbank) of FASN, FABP4, and SCD of the Hereford bovine breed. The Sensitivity was set on the Medium Sensitivity/Fast and the Fine-Tuning (iterate up to 5 times) option was selected to improve the results by aligning reads to each other in addition to the reference sequence. Major allele frequencies from the next-generation sequencing data based on observed and expected genotypes were computed using the Hardy–Weinberg equilibrium principle.

### 2.7. Statistical Analyses

All statistical analyses of the associations between identified SNP of the SCD, FASN, and FABP4 genes with IMF, FMP, and fatty acid composition were performed using R software v.4.0.2 (R Foundation for Statistical Computing, Vienna, Austria). Descriptive summaries were presented as medians and inter-quartile ranges. Spearman correlations were used to estimate the relationships between SNP and meat quality traits (fatty acids, FMP, and IMF). Fatty acids, IMF, and FMP were analyzed as dependent variables using a non-parametric analysis of variance (Kruskal-Wallis test).

$$Y_{ij}=\mu +\tau_{i}+\epsilon_{ij}$$

where $\gamma_{ij}$ is the quantitative phenotype (meat quality traits), μ is the overall mean, $\tau_{i}\;$ is the SNP effect and *∈_ij_* is the random error. Differences between medians were compared using the Tukey-adjusted multiple comparisons test. The threshold for significance was set at *p* < 0.05.

## 3. Results

The candidate genes SCD, FASN, and FABP4 were targeted for this study because of their known physiological roles in intramuscular fat deposition and fatty acid composition. SNP identification in the sequences of SCD, FASN, and FABP4 lipogenic genes was made possible due to the successful primer design in Geneious Prime software, as depicted in Table 1 and corroborated by the gel images of the PCR products shown in Figure 1, Figure 2 and Figure 3.

### 3.1. SCD, FASN, and FABP Variants and Genotypes

This study used the reference sequence extracted from the NCBI database for Hereford beef cattle, resulting in 12 identified SCD SNP with major allele frequencies ranging from 0.53 to 0.97, as shown in Table 2. A further identification of 18 SNP of the FASN gene with major allele frequencies ranging from 0.50 to 1.00 (Table 3) and five SNP in the FABP4 gene with major allele frequencies ranging from 0.50 to 0.98 is depicted in Table 4. It is evident from Table 2 that the Wagyu was heterozygous in SNP g.21267406 T>C, g.21267451 C>A and g.21267897 C>T, but homozygous in nine other SNP (g.21268157 G>A; g.21269020 A>G; g.21269495 G>C; g.21270740 A>G; g.21271264 C>A; g.21271726 C>G; g.21272247 A>G; g.21272423 C>T; g.21275852 C>A). Angus and Hereford were homozygous in most SCD SNP, except for SNP g.21268157 G>A (Table 2). As depicted in Table 3, Wagyu was homozygous, while Angus was heterozygous in ten FASN SNP (g.50783867 C>A; g.50787010 A>G; g.50787138 A>G; g.50788320 C>T; g.50788576 T>C; g.50789409 T>C; g.50789883 T>C; g.50792348 C>T; g.50793358 A>G; g.50794100 T>C). The Hereford was heterozygous at five SNP loci (g.50787010 A>G; g.50789409 T>C; g.50789883 T>C; g.50792348 C>T; g.50794100 T>C). For the FABP4 gene, Angus was heterozygous in SNP g.44680268 C>G; whereas Hereford and Wagyu breeds were homozygous (Table 4).

### 3.2. Correlations between SNP, FMP, IMF, and Health-Beneficial n-3 LC-PUFA

**Figure 4:**
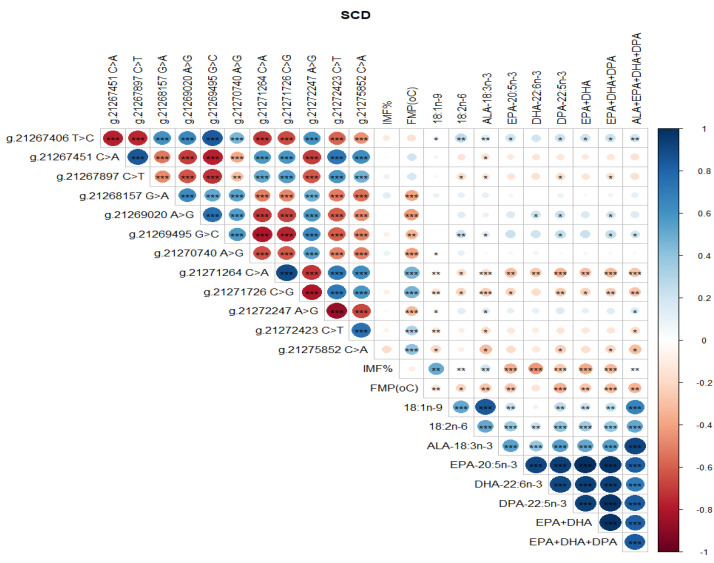
Spearman’s correlations between SCD gene SNP loci, IMF, FMP, and fatty acid profiles of Bowen Genetics Forest Pastoral Angus, Hereford, and Wagyu beef cattle breeds. (* *p* < 0.05, ** *p* < 0.01; *** *p* < 0.001). Dark blue indicates a strong positive relationship, and dark red shows a strong negative relationship.

**Figure 5:**
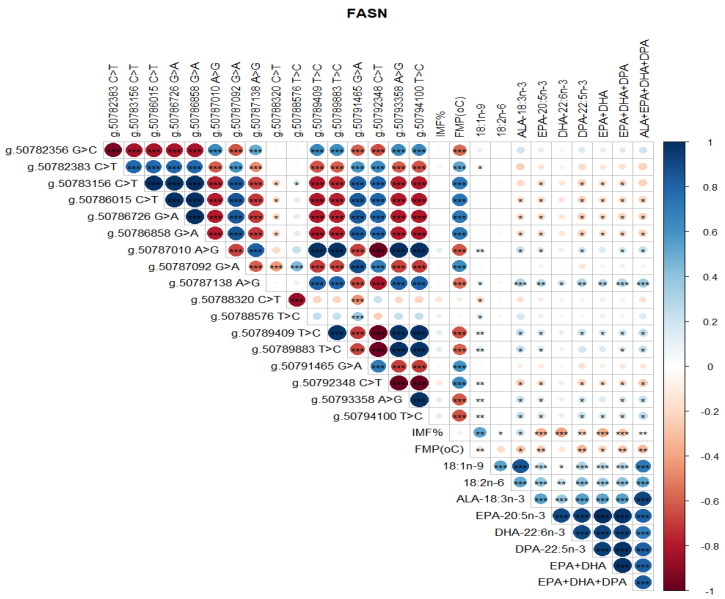
Spearman’s correlations between FASN gene SNP loci, IMF, FMP, and fatty acid profiles of Bowen Genetics Forest Pastoral Angus, Hereford, and Wagyu beef cattle breeds. (* *p* < 0.05, ** *p* < 0.01; *** *p* < 0.001). Dark blue indicates a strong positive relationship, and dark red shows a strong negative relationship.

**Figure 6:**
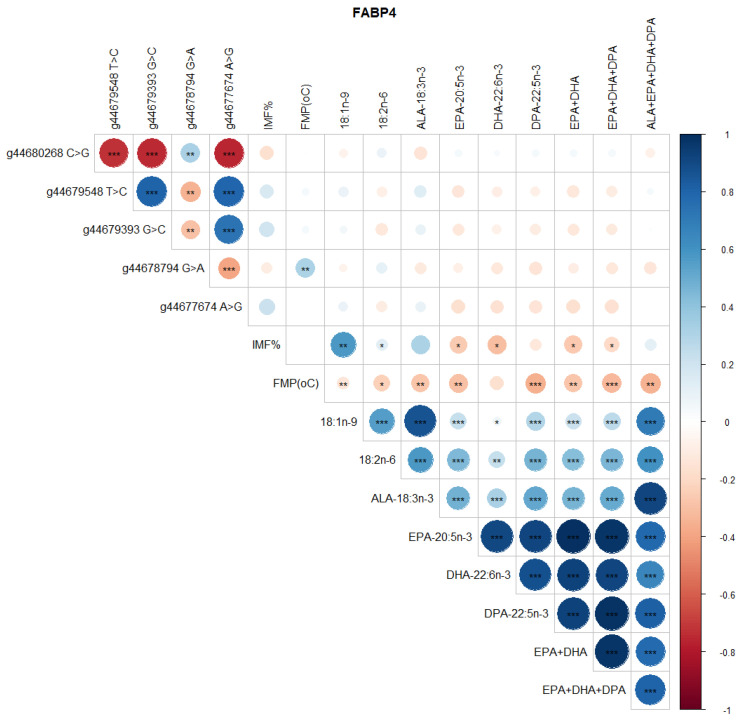
Spearman’s correlations between FABP4 gene SNP loci, IMF, FMP, and fatty acid profiles of Bowen Genetics Forest Pastoral Angus, Hereford, and Wagyu beef cattle breeds. (* *p* < 0.05, ** *p* < 0.01; *** *p* < 0.001). Dark blue indicates a strong positive relationship, and dark red shows a strong negative relationship.

Significant correlations were detected between SCD SNP, meat quality traits (IMF, FMP), and health-beneficial n-3 LC-PUFA (Figure 4). Notably, positive correlations between SNP g.21267406 T>C and g.21269495 G>C, g.21267451 C>A and g.21267897 C>T, and g.21271264 C>A and g.21271726 C>G were observed (Figure 4). There were negative correlations between SNP g.21267406 T>C and g.21267451 C>A, g.21271726 C>G and g.21272247 A>G, and g.21272247 A>G and g.21272423 C>T (Figure 4). There were positive correlations between SCD SNP g.21267406 T>C and 18:1n-9, 18:2n-6 (LA, linoleic acid), ALA, EPA, DHA, DPA, total EPA+DHA+DPA, and total ALA + EPA + DHA + DPA (*p* < 0.05). SCD SNP g.21271264 C>A and g.21271726 C>G were positively correlated with FMP (*p* < 0.001), but negatively correlated with health-beneficial n-3 LC-PUFA (ALA, EPA, DHA, DPA, total EPA + DHA, total EPA + DHA + DPA, total ALA + EPA + DHA + DPA), oleic acid, and LA. Positive correlations between IMF and 18:1n-9, and negative correlations of FMP with IMF and all of the n-3 LC-PUFA and their summations were evident (Figure 4). Positive correlations (*P* < 0.05) were observed between FASN SNP g. 50787138 A>G and oleic 18:1n-9, ALA, EPA, DHA, DPA, EPA + DHA, EPA + DHA + DPA, and ALA + EPA + DHA + DPA (Figure 5). However, this SNP was negatively correlated with FMP (Figure 5). In Figure 6, FABP4 SNP g.44679548 T>C and g.44679393 G>C, and g.44679548 T>C and g.44677674 A>G were all positively correlated, while negative correlations were observed between SNP g.44680268 C>G and g.44679548 T>C, g.44680268 C>G and g.44679393 G>C, and g.44680268 C>G and g.44677674 A>G. A positive correlation between FABP4 SNP g.44678794 G>A and FMP was observed (Figure 6).

### 3.3. Associations between SNP and IMF, FMP, and Health-Beneficial n-3 LC-PUFA

Two SCD SNP g.21267406 T>C and g.21271264 C>A were significantly associated with health-beneficial n-3 LC-PUFA and meat quality traits (Table 5 and Table 6). Other significant associations observed were between SCD SNP g.21267406 T>C and LA (*p* = 0.033), ALA (*p* = 0.003), EPA (*p* = 0.047), DHA (*p* = 0.009), DPA (*p* = 0.012), EPA + DHA (*p* = 0.032), EPA + DHA + DPA (*p* = 0.012), and ALA + EPA + DHA + DPA (*p* = 0.002). SCD SNP g.21271264 C>A was significantly associated with FMP (*p* = 0.000), oleic acid (*p* = 0.016), ALA (*p* = 0.003), EPA (*p* = 0.022), DHA (*p* = 0.009), DPA (*p* = 0.003), EPA + DHA (*p* = 0.015), EPA + DHA + DPA (*p* = 0.003), and ALA + EPA + DHA + DPA (*p* = 0.004). Table 7 shows that FASN SNP g.50787138 A>G was significantly associated with FMP (*p* = 0.000), oleic acid (*p* = 0.021), ALA (*p* = 0.001), EPA (*p* = 0.008), DHA (*p* = 0.040), DPA (*p* = 0.007), EPA + DHA (*p* = 0.007), EPA + DHA + DPA (*p* = 0.004), and ALA + EPA + DHA + DPA (*p* = 0.002). As demonstrated in Table 8, FABP4 SNP g.44678794 G>A was significantly associated with FMP (*p* = 0.011).

### 3.4. Tukey-Adjusted Multiple Comparison Tests for Significant SNP, FMP, IMF, and Health-Beneficial n-3 LC-PUFA

**Figure 7:**
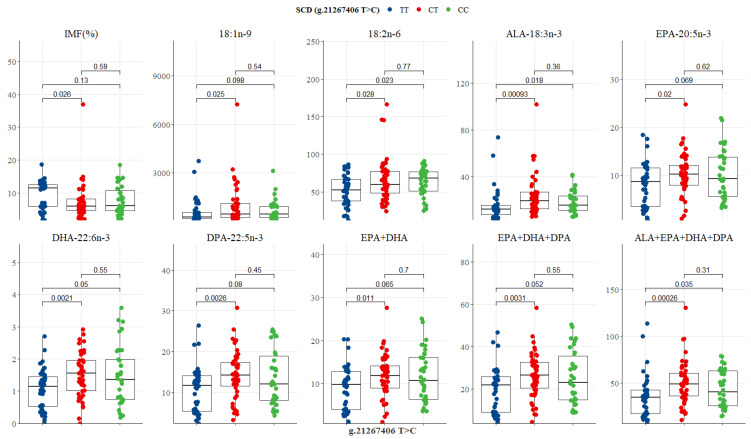
Tukey-adjusted multiple comparisons between an SCD SNP locus with IMF and fatty acid profiles of Bowen Genetics Forest Pastoral Angus, Hereford, and Wagyu. Each box plot tested the mean IMF, FMP, and fatty acids in TT versus CT, TT versus CC, and CT versus CC genotypes. Significant level set at (*p* < 0.05).

The SCD SNP g.21267406 T>C showed significant differences between genotypes, where the heterozygous CT genotype had the lowest IMF (6%), but the highest content of oleic acid, ALA, EPA, DHA, DPA, EPA + DHA, EPA + DHA + DPA, and ALA + EPA + DHA + DPA (453.9, 19.44, 10.18, 1.56, 14.19, 11.89, 26.41, and 49.05 mg/100g, respectively), while CC was intermediate and TT lowest in n-3 LC-PUFA (Table 5 and Figure 7). CC genotype had the highest LA (68.07 mg/100g), and the TT genotype had the highest IMF (11.46%) as depicted in Table 5 and Figure 7. Significant differences were observed between CT and TT genotypes for IMF (*p* = 0.026), oleic acid (*p* = 0.025), LA (*p* = 0.028), ALA (*p* = 0.000), EPA (*p* = 0.020), DHA (*p* = 0.002), DPA (*p* = 0.003), EPA + DHA (*p* = 0.011), EPA + DHA + DPA (*p* = 0.003), and ALA + EPA + DHA + DPA (*p* = 0.000) (Figure 7). Differences between TT and CC genotypes were evident in the compositions of LA (*p* = 0.023), ALA (*p* = 0.018), DHA (*p* = 0.050), EPA + DHA + DPA (*p* = 0.052), and ALA + EPA + DHA + DPA (*p* = 0.035), as shown in Figure 7. No difference was seen between CT and CC genotypes (Figure 7).

**Figure 8:**
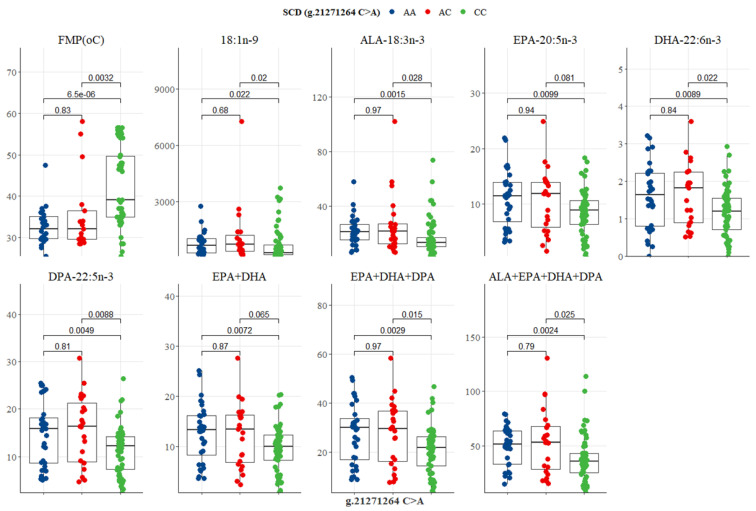
Tukey-adjusted multiple comparisons between an SCD SNP locus with FMP, IMF, and fatty acid profiles of Bowen Genetics Forest Pastoral Angus, Hereford, and Wagyu. Each box plot tested the mean IMF, FMP, and fatty acid in AA versus AC, AA versus CC, and AC versus CC genotypes. Significant level set at (*p* < 0.05).

The SCD g.21271264 C>A SNP showed significant variation in FMP and n-3 LC-PUFA. The CC genotype had the highest FMP (39.66 °C), but the lowest oleic acid, ALA, EPA, DPA, EPA + DHA, EPA + DHA + DPA, and ALA + EPA + DHA + DPA (273.66, 13.66, 8.66, 12.66, 9.66, 21.66, and 35.66 mg/100g, respectively), as depicted in Table 6 and Figure 8. EPA + DHA + DPA for genotypes AA (29.66 mg/100g) and AC (29.66 mg/100g) were in close proximity to the recommended ‘source’ level of 30 mg/100g in Australia and New Zealand (Table 6). There were significant differences between CC versus AC genotypes, and CC versus AA genotypes for FMP, oleic acid, ALA, EPA, DHA, DPA, EPA + DHA, EPA+DHA+DPA, and ALA + EPA + DHA + DPA, as shown in Figure 8. No significant difference between genotypes AA and AC was observed in eating quality traits and fatty acid summations (Figure 8).

**Figure 9:**
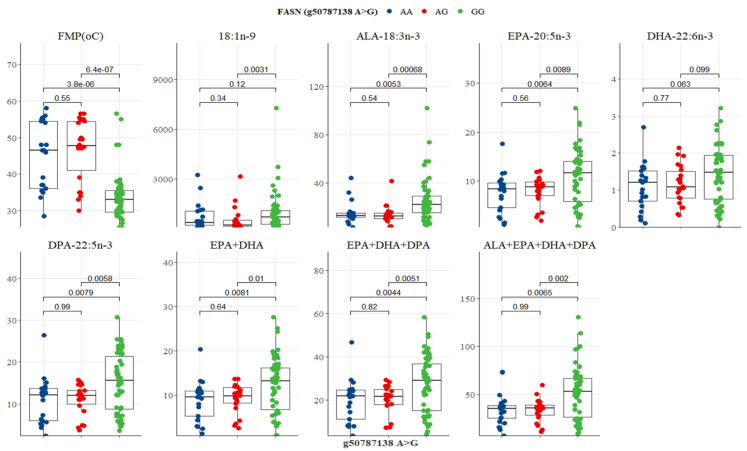
Tukey-adjusted multiple comparisons between a FASN SNP locus with FMP, IMF, and fatty acid profiles of Bowen Genetics Forest Pastoral Angus, Hereford, and Wagyu. Each box plot tested the median IMF, FMP, and fatty acid in AA versus AG, AA versus GG, and AG versus GG genotypes. Significant level set at (*p* < 0.05).

**Figure 10:**
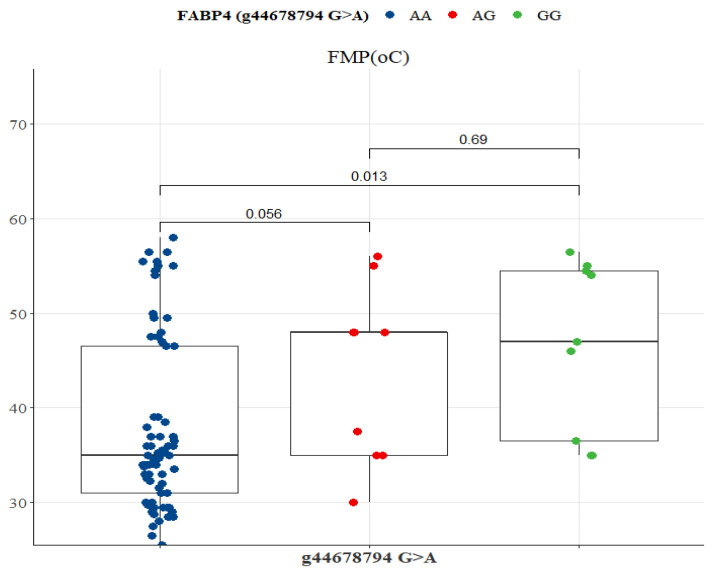
Tukey-adjusted multiple comparisons between a FABP4 SNP locus with FMP of Bowen Genetics Forest Pastoral Angus, Hereford, and Wagyu. The box plot tested the median FMP of AA versus AG, AA versus GG, and AG versus GG genotypes. Significant level set at (*p* < 0.05).

FASN SNP g.50787138 A>G was associated with variations in FMP, oleic acid, ALA, EPA, DPA, EPA + DHA, EPA + DHA + DPA, and ALA + EPA + DHA + DPA (Figure 9). GG genotype had the lowest FMP (33 °C), but the highest n-3 LC-PUFA compared to AA and AG genotypes (Table 7). Significant differences existed between GG versus AG and GG versus AA genotypes for all variables (*p* < 0.05; Figure 9). There was no significant difference between AA and AG genotypes (Figure 9). In the FABP SNP g.44678794 G>A, only FMP differed between genotypes. AA genotype had the lowest FMP (35 °C), but both GG and AG were at 45 °C (Table 8). AA genotype differed significantly from AG (*p* = 0.056) and GG (*p* = 0.013) genotypes (Figure 10).

## 4. Discussion

The bioavailability of supplemented omega-3 fatty acids is significantly affected by biohydrogenation in ruminants [51] due to the conversion of unsaturated fatty acids to SFA moieties, which are absorbed in the small intestine and deposited in edible beef muscle tissues [52]. Genomic selection using breed-specific DNA markers may considerably improve health-promoting n-3 LC-PUFA and meat eating quality traits in beef cattle and the accuracy of selection [53].

### 4.1. SCD Gene Polymorphism

The SCD gene plays a role in the biosynthesis of monounsaturated fatty acids (MUFA) from stearic and palmitic acids, by introducing a double bond between carbon atoms Δ^9^ and Δ^10^ [54]. SCD enzyme activity has been shown to be positively correlated with unsaturated fatty acid content and marbling in Angus [55], Hereford [31], Japanese Black [56], Jersey [57], and Italian Holstein [58] cattle. Most studies have focused on the association between SCD SNP and oleic acid, but in the present study, the finding that SCD SNP were significantly associated with health-beneficial n-3 LC-PUFA and meat quality traits (Table 5 and Table 6, Figure 7 and Figure 8) complements previous reports [31,44,54,59]. The fact that beef cattle breeds with heterozygous genotypes CT and AC in the SCD SNP g.21267406 T>C and g.21271264 C>A produced the highest oleic acid, ALA, EPA, DHA, DPA compared to CC, TT and AA genotypes (Table 5 and Table 6), represents a genetic divergence between the three beef cattle breeds that provides insights into the role of the SCD gene in the metabolism of EPA, DHA, and DPA. This underscores the potential of SCD SNP in marker-assisted selection to improve the composition of health-promoting n-3 LC-PUFA and meat eating quality attributes in Australia’s beef cattle grazing systems.

### 4.2. FASN Gene Polymorphism

Fatty acid synthase (FASN) catalyzes the de novo synthesis of palmitic and stearic acids [19,60]. Abe et al. [61] and Barton et al. [62] reported three FASN SNP loci (g.16024G>A, 16039T>C, and g.17924A>G), in exons 34 and 39, that caused an amino acid substitution of threonine to alanine in the thioesterase domain. These FASN polymorphisms were significantly associated with oleic, palmitic, and palmitoleic acids [19,62,63]. Yokota et al. [64] reported an additive effect of SNP g.16039T>C on several SFA and MUFA in the intramuscular fatty acids of Japanese Black cattle. The impact of FASN SNP g.17924A>G on fatty acid composition was also reported in Angus cattle [19], Hereford [63], and crossbred beef steers [65]. FASN SNP g.12870, g.13126, g.15532, g.16907, and g.17924 were reported to be associated with intramuscular fatty acids in Korean cattle. Results of the present study also demonstrated that polymorphisms in the FASN SNP g.50787138 A>G detected in intron 21 have positive associations with FMP, oleic acid, EPA, DHA, DPA, and total n-3 LC-PUFA, thus suggesting a synonymous mutation. Previous studies reported non-synonymous mutations where an amino acid substitution of threonine to alanine was observed [61,62]. In the present study, the finding that GG genotype had the lowest FMP and the highest oleic acid, ALA, EPA, DHA, DPA, and EPA+DHA+DPA compared to AG and AA genotypes (Table 7 and Figure 9) confirms that the g.50787138 A>G SNP can effectively be used as a selection marker due to its significant association with health-promoting n-3 LC-PUFA metabolism in pasture-based beef cattle.

### 4.3. FABP4 Gene Polymorphism

The fatty acid-binding protein-4 (FABP4) plays a role in the absorption, transport, and metabolism of long-chain fatty acids [66,67,68]. Previous studies reported an association between FABP4 polymorphism and carcass characteristics [35] and fat deposition [66,67]. In Japanese Black cattle, Hoashi et al. [69], reported that genotype 174V of the FABP4 gene was significantly associated with 16:1 and 18:2. FABP4 SNP g.2834C>G and g.3631G>A were associated with marbling [41]. Shin et al. [42] found a significant correlation of FABP4 SNP g.3691G>A with marbling and meat quality traits in Korean cattle. Other studies evaluated the associations of FABP4 SNP with carcass characteristics, 14:0, 16:1, 16:1n-7, LA, and oleic acid [62,67,69,70]. In the present study, FABP4 SNP g.44678794 G>A was significantly correlated with FMP, where the AA genotype had the lowest fat melting point of 35 °C (Table 8). This suggests that polymorphisms in intron 1 of the FABPA gene controlling fat melting point could be influential markers in specifically selecting for healthy polyunsaturated fatty acids in pasture-based Angus, Hereford, and Wagyu since genetic markers are often breed-specific and may not be extrapolated to other cattle breeds [46,62].

## 5. Conclusions

This study identified SCD SNP g.21267406 T>C and g.21271264 C>A in introns 2 and 4, FASN SNP g.50787138 A>G in intron 21 and FABP4 SNP g.44678794 G>A in intron 1 that were significantly associated with FMP, IMF, and health-beneficial n-3 LC-PUFA fatty acids. These results, taken together, provide an opportunity for SNP marker-assisted selection for improving meat eating quality and health-promoting n-3 LC-PUFA traits in Australian pasture-based Bowen Genetics Forest Pastoral beef cattle while they are young and still alive. As these data are actually laboratory tested, individualized, and customized to each cattle breed, as opposed to estimated breeding values based on herd averages, they increase the precision and accuracy of the selection program. They also enable Australian beef farmers to make early decisions in quantifying the genetic worth of their animals for meat eating quality and health-beneficial n-3 LC-PUFA based on muscle biopsy sampling and the development of a novel selection index for beef quality in their breeding program.

## Figures and Tables

**Table 1:** Primer pairs and sequences for SCD, FASN, and FABP4 genes for polymerase chain reaction assays.

Gene Symbol	Primer	Primer Sequence	Length	T_a_ (°C)	Amplification Loci
SCD1	Forward	GGAAGAAGACATCCGCCCTGAAAT	24	60	Exons: 2–6 Introns: 2–4
	Reverse	TCCAGGCGCCAAATAGAGTCTCTA	24	60	
SCD2	Forward	AGAGTGGTGGTCATGCACAAACTT	24	60	
	Reverse	CATACAGTGCCAATCTCGCTTCCT	24	60	
SCD3	Forward	AGAGTGGTGGTCATGCACAAACTT	24	60	
	Reverse	ACCAAATAGATCCCCTCAGAGGCA	24	60	
SCD4	Forward	ATGAGCCACACTGTGAACAAACCT	24	60	
	Reverse	AGGCTTGCCTGTCCAGAAAAAGAA	24	60	
FASN1	Forward	TTGAGCTTCTGAGTATGATGGGAG	24	68	Exons: 9–42 Introns: 8–41
	Reverse	GTTGAGGGAGGCATAATAGATGGT	24	68	
FASN2	Forward	CTATAAGATCGGTGAGTCCTTGCA	24	68	
	Reverse	GCCAGGGAGCTGTGAATAATACTA	24	68	
FABP41	Forward	GCTAAGACTGCCTGTATGTTCCCC	24	60	Exons: 2 and 3 Introns: 1 and 2
	Reverse	GGGCGATTGTCTATTTCTCTAGGT	24	60	

SCD: Stearoyl-CoA Desaturase, *FASN*: Fatty Acid Synthase, *FABP4*: Fatty Acid Binding Protein. T_a_: Annealing Temperature.

**Table 2:** SCD gene SNP in Bowen Genetics Forest Pastoral Angus, Hereford, and Wagyu beef cattle.

SNP Locus, Cattle Breeds, Reference Genotype, and Location (Major Allele Frequencies in Brackets)					
SNP Locus	Wagyu	Angus	Hereford	Genotype	Location
g.21267406 T>C	CT (0.55)	T (0.68)	T (0.61)	TT	Intron 2
g.21267451 C>A	AC (0.57)	C (0.73)	CC (0.73)	CC	Intron 2
g.21267897 C>T	TC (0.56)	C (0.68)	C (0.73)	CC	Intron 2
g.21268157 G>A	G (0.86)	AG (0.55)	A (0.59)	GG	Intron 2
g.21269020 A>G	G (0.74)	A (0.75)	A (0.68)	AA	Intron 3
g.21269495 G>C	C (0.55)	G (0.75)	G (0.80)	GG	Intron 3
g.21270740 A>G	G (0.69)	A (0.87)	A (0.89)	AA	Intron 4
g.21271264 C>A	A (0.59)	C (0.95)	C (0.95)	CC	Intron 4
g.21271726 C>G	G (0.54)	C (0.97)	C (0.95)	CC	Intron 4
g.21272247 A>G	A (0.59)	A (0.95)	A (0.93)	AA	Exon 5
g.21272423 C>T	C (0.58)	C (0.93)	C (0.93)	CC	Exon 5
g.21275852 C>A	A (0.53)	C (0.93)	C (0.95)	CC	Exon 5

SNP: Single nucleotide polymorphisms.

**Table 3:** FASN gene SNP in Bowen Genetics Forest Pastoral Angus, Hereford, and Wagyu beef cattle.

SNP Locus, Cattle Breeds, Reference Genotype, and Location (Major Allele Frequencies in Brackets)					
SNP Locus	Wagyu	Angus	Hereford	Genotype	Location
g.50782356 G>C	C (0.70)	G (1.00)	G (0.95)	GG	Intron 8
g.50782383 C>T	T (0.92)	C (1.00)	C (0.95)	CC	Intron 8
g.50783156 C>T	T (0.92)	C (1.00)	C (0.93)	CC	Exon 10
g.50783867 C>A	A (0.94)	AC (0.52)	C (0.73)	CC	Intron 11
g.50786015 C>T	T (0.93)	C (1.00)	C (0.89)	CC	Intron 18
g.50786726 G>A	A (0.93)	G (1.00)	G (0.91)	GG	Exon 21
g.50786858 G>A	A (0.93)	G (1.00)	G (0.93)	GG	Exon 21
g.50787010 A>G	G (1.00)	GA (0.59)	GA (0.66)	AA	Intron 21
g.50787092 G>A	A (0.82)	G (1.00)	G (0.89)	GG	Intron 21
g.50787138 A>G	G (0.94)	GA (0.62)	A (0.68)	AA	Intron 21
g.50788320 C>T	C (0.76)	TC (0.53)	C (0.77)	CC	Exon 23
g.50788576 T>C	T (0.72)	CT (0.60)	T (0.77)	TT	Exon 24
g.50789409 T>C	C (1.00)	CT (0.52)	CT (0.67)	TT	Exon 27
g.50789883 T>C	C (1.00)	CT (0.50)	CT (0.65)	TT	Intron 28
g.50791465 G>A	A (0.77)	G (1.00)	G (0.95)	GG	Exon 34
g.50792348 C>T	T (1.00)	TC (0.50)	TC (0.70)	CC	Exon 37
g.50793358 A>G	G (1.00)	GA (0.50)	GA (0.70)	AA	Exon 39
g.50794100 T>C	C (1.00)	CT (0.52)	CT (0.69)	TT	Exon 42

SNP: Single nucleotide polymorphisms.

**Table 4:** FABP4 gene SNP in Bowen Genetics Forest Pastoral Angus, Hereford, and Wagyu beef cattle.

SNP Locus, Cattle Breeds, Reference Genotype, and Location (Major Allele Frequencies in Brackets)					
SNP Locus	Wagyu	Angus	Hereford	Genotype	Location
g.44680268 C>G	G (0.56)	GC (0.50)	C (0.60)	CC	Exon 3
g.44679548 T>C	C (0.55)	C (0.55)	T (0.55)	TT	Exon 2
g.44679393 G>C	C (0.51)	C (0.54)	G (0.68)	GG	Intron 1
g.44678794 G>A	A (0.98)	A (0.76)	A (0.69)	GG	Intron 1
g.44677674 A>G	G (0.59)	A (0.54)	A (0.58)	AA	Intron 1

SNP: Single nucleotide polymorphisms.

**Table 5:** Descriptive summaries of the associations between SCD SNP locus g.21267406 T>C and genotypes with intramuscular fat (IMF), fat melting point (FMP), and fatty acid composition in Bowen Genetics Forest Pastoral Angus, Hereford, and Wagyu beef cattle presented as median (inter-quartile range).

Variable	SNP g.21267406 T>C				
	Overall	TT	CT	CC	*p*-Value
IMF (%)	6.29 (4.98–12)	11.46 (5.91–12.63)	6 (4.76–8.16)	6.14 (4.62–10.78)	0.0744
FMP (°C)	35.38 (32.22–47.5)	36 (34.5–48)	34.75 (31.75–46.12)	34.5 (30–38.25)	0.2105
Oleic acid-18:1n-9	384.17 (198.1–946.84)	249.25 (166.24–560.71)	453.9 (221.45–1102.78)	436.75 (227.41–947.61)	0.0614
LA-18:2n-6	58.26 (43.06–75.75)	52.61 (37.82–66.65)	59.85 (48.61–77.17)	68.07 (51.13–76.98)	0.0334
ALA-18:3n-3	15.08 (11.43–23.98)	12.54 (7.92–15.76)	19.44 (12.7–26.92)	15.91 (11.43–23.66)	0.0025
EPA-20:5n-3	9.29 (5.53–11.97)	8.65 (3.43–11.47)	10.18 (7.95–12.06)	9.21 (5.49–13.79)	0.0466
DHA-22:6n-3	1.32 (0.78–1.84)	1.15 (0.53–1.45)	1.56 (1.02–1.96)	1.36 (0.75–1.98)	0.0087
DPA-22:5n-3	12.74 (7.62–16.23)	11.68 (5.4–14.16)	14.19 (11.62–17.38)	12.11 (8.05–18.93)	0.0119
EPA+DHA	10.65 (6.37–13.77)	9.74 (3.9–12.83)	11.89 (8.98–14.1)	10.7 (6.35–16.06)	0.0322
EPA+DHA+DPA	23.7 (14.8–30.34)	21.8 (8.98–25.77)	26.41 (20.36–32.53)	23.03 (14.68–35.46)	0.0121
ALA+EPA+DHA+DPA	38.17 (25.71–57.11)	34.91 (17.16–42.58)	49.05 (36.28–60.57)	40.84 (25.6–63.3)	0.0016

SNP: single nucleotide polymorphism, IMF: intramuscular fat, FMP: fat melting point, LA: linoleic acid, ALA: alpha-linolenic acid, EPA: eicosapentaenoic acid, DHA: docosahexaenoic acid, DPA: docosapentaenoic acid. Non-parametric ANOVA (Kruskal–Wallis test) *p*-value.

**Table 6:** Descriptive summaries of the associations between SCD SNP locus g.21271264 C>A and genotypes with intramuscular fat (IMF), fat melting point (FMP), and fatty acid composition in Bowen Genetics Forest Pastoral Angus, Hereford, and Wagyu beef cattle presented as median (inter-quartile range).

Variable	SNP g.21271264 C>A				
	Overall	AA	AC	CC	*p*-Value
IMF (%)	6.66 (4.98–12)	7.66 (4.37–12.62)	6.66 (4.61–8.16)	6.66 (5.48–12.12)	0.3425
FMP (°C)	35.66 (32.22–47.5)	32.66 (29.69–35.12)	32.66 (29.5–36.5)	39.66 (35–49.62)	0.0001
Oleic acid-18:1n-9	384.66 (198.1–946.84)	649.66 (264.47–1013.27)	702.66 (342.28–1215.38)	273.66 (162.07–694.26)	0.0156
LA-18:2n-6	58.66 (43.06–75.75)	68.66 (53.96–76.2)	58.66 (48.27–75.88)	57.66 (39.19–75.69)	0.1852
ALA-18:3n-3	15.66 (11.43–23.98)	21.66 (15.17–26.38)	21.66 (12.23–27)	13.66 (10.37–16.81)	0.0028
EPA-20:5n-3	9.66 (5.53–11.97)	11.66 (6.84–13.9)	11.66 (5.78–13.92)	8.66 (6.29–10.59)	0.0223
DHA-22:6n-3	1.66 (0.78–1.84)	1.66 (0.79–2.21)	1.66 (0.89– 2.24)	1.66 (0.7–1.54)	0.0089
DPA-22:5n-3	12.66 (7.62–16.23)	15.66 (8.68–18.18)	16.66 (8.85–21.25)	12.66 (7.3–14.15)	0.0032
EPA + DHA	10.66 (6.37–13.77)	13.66 (8.23–16.15)	13.66 (6.72–16.16)	9.66 (7.27–12.21)	0.0152
EPA + DHA + DPA	23.66 (14.8–30.34)	29.66 (17.02–33.66)	29.66 (16.21–36.8)	21.66 (14.57–26.41)	0.0032
ALA + EPA + DHA + DPA	38.66 (25.71–57.11)	51.66 (33.07–63.46)	53.66 (28.39–67.69)	35.66 (25.45–42.79)	0.0039

SNP: single nucleotide polymorphism, IMF: intramuscular fat, FMP: fat melting point, LA: linoleic acid, ALA: alpha-linolenic acid, EPA: eicosapentaenoic acid, DHA: docosahexaenoic acid, DPA: docosapentaenoic acid. Non-parametric ANOVA (Kruskal–Wallis test) *p*-value.

**Table 7:** Descriptive summaries of the associations between FASN SNP locus g. 50787138 A>G and genotypes with intramuscular fat (IMF), fat melting point (FMP), and fatty acid composition in Angus, Hereford, and Wagyu beef cattle breeds presented as median (inter-quartile range).

Variable	SNP g. 50787138 A>G				
	Overall	AA	AG	GG	*p*-Value
IMF (%)	6.29 (2.98–12)	5.96 (2.02–11.62)	5.96 (2.67–11.65)	7.17 (2.03–12.27)	0.9507
FMP (°C)	35.38 (2.22–47.5)	46.5 (2.36–54.5)	47.75 (2.41–54.38)	33 (2.5–35.38)	0.0001
Oleic acid-18:1n-9c	384.17 (2.1–946.84)	301.52 (2.36–891.68)	221.67 (2.5–485.2)	514.21 (2.92–1058.96)	0.02118
LA-18:2n-6	58.26 (2.06–75.75)	57.7 (2.86–75.84)	50.91 (2.88–71.44)	60.52 (2.73–75.81)	0.2839
ALA-18:3n-3	15.08 (2.43–23.98)	13.43 (2.7–14.51)	12.32 (2.36–14.65)	21.45 (2.24–28.49)	0.0004
EPA-20:5n-3	9.29 (2.53–11.97)	8.36 (2.52–9.77)	8.78 (2.12–9.7)	11.54 (2.46–13.83)	0.0083
DHA-22:6n-3	1.32 (2.78–1.84)	1.24 (2.76–1.54)	1.05 (2.79–1.46)	1.48 (2.81–1.99)	0.0396
DPA-22:5n-3	12.74 (2.62–16.23)	12.1 (2.22–13.54)	11.84 (2.44–13.14)	15.08 (2.96–18.81)	0.0072
EPA+DHA	10.65 (2.37–13.77)	9.54 (2.21–11.0)	9.58 (2.18–11.45)	13.1 (2.33–15.97)	0.0071
EPA+DHA+DPA	23.7 (2.8–30.34)	21.83 (2.88–24.86)	21.62 (2.84–24.78)	28.75 (2.02–35.46)	0.0039
ALA+EPA+DHA+DPA	38.17 (2.71–57.11)	35.4 (2.69–39.48)	35.79 (2.57–38.36)	51.98 (2.49–63.46)	0.0016

SNP: single nucleotide polymorphism, IMF: intramuscular fat, FMP: fat melting point, LA: linoleic acid, ALA: alpha-linolenic acid, EPA: eicosapentaenoic acid, DHA: docosahexaenoic acid, DPA: docosapentaenoic acid. Non-parametric ANOVA (Kruskal–Wallis test) *p*-value.

**Table 8:** Descriptive summaries of the associations between FABP4 SNP locus g.44678794 G>A and genotypes with intramuscular fat (IMF), fat melting point (FMP), and fatty acid composition in Bowen Genetics Forest Pastoral Angus, Hereford, and Wagyu beef cattle presented as median (inter-quartile range).

Variable	SNP g.44678794 G>A				
	Overall	AA	AG	GG	*p*-Value
IMF (%)	6.25 (4.98–12)	6.75 (4.79–11.95)	5.95 (5.52–10.05)	5.95 (4.05–6.85)	0.4059
FMP (°C)	35.5 (32.22–47.5)	35 (31–46.5)	45 (35–48)	45 (36.5–54.5)	0.0111
Oleic acid-18:1n-9c	384.5 (198.1–946.84)	415.5 (212.81–939.11)	235.5 (124.18–612.33)	271.5 (198.92–415.96)	0.2612
LA-18:2n-6	58.5 (43.06–75.75)	58.5 (39.12–76.3)	56.5 (51.28–69.1)	61.5 (57.09–75.34)	0.7161
ALA-18:3n-3	15.5 (11.43–23.98)	15.5 (11.46–24.21)	14.5 (11.81–14.94)	15 (11.97–19.62)	0.7559
EPA-20:5n-3	9.25 (5.53–11.97)	9.45 (5.42–12.34)	8.55 (7.95–11.04)	9.25 (8.11–11.24)	0.9722
DHA-22:6n-3	1.35 (0.78–1.84)	1.45 (0.75–1.96)	1.25 (1.08–1.32)	1.15 (0.98–1.48)	0.5943
DPA-22:5n-3	12.5 (7.62–16.23)	12.5 (7.27–17.02)	12.5 (11.3–14.85)	12.5 (11.02–13.16)	0.7451
EPA+DHA	10.5 (6.37–13.77)	11.5 (6.21–14.13)	9.85 (8.87–12.43)	10.5 (9.36–12.19)	0.9100
EPA+DHA+DPA	23.5 (14.8–30.34)	24.5 (13.49–30.98)	22.5 (20.41–27.3)	21.5 (19.55–25.42)	0.7784
ALA+EPA+DHA+DPA	38.5 (25.71–57.11)	41.5 (25.46–59.66)	36.5 (32.36–41.51)	37.5 (32.86–46.99)	0.7167

SNP: single nucleotide polymorphism, IMF: intramuscular fat, FMP: fat melting point, LA: linoleic acid, ALA: alpha-linolenic acid, EPA: eicosapentaenoic acid, DHA: docosahexaenoic acid, DPA: docosapentaenoic acid. Non-parametric ANOVA (Kruskal–Wallis test) *p*-value.

## Data Availability

The data presented in this study are available from the corresponding author on request.
